# Comment on “Glass Transition, Crystallization of Glass-Forming Melts, and Entropy” *Entropy* 2018, *20*, 103

**DOI:** 10.3390/e20090703

**Published:** 2018-09-13

**Authors:** Edgar D. Zanotto, John C. Mauro

**Affiliations:** 1 Department of Materials Engineering, Center for Research, Technology and Education in Vitreous Materials, Federal University of São Carlos, São Paulo 13.565-905, Brazil; 2 Department of Materials Science and Engineering, The Pennsylvania State University, University Park, PA 16802, USA

**Keywords:** glass, thermodynamics, entropy, relaxation, viscosity, statistical mechanics

## Abstract

In a recent article, Schmelzer and Tropin [*Entropy* **2018**, *20*, 103] presented a critique of several aspects of modern glass science, including various features of glass transition and relaxation, crystallization, and the definition of glass itself. We argue that these criticisms are at odds with well-accepted knowledge in the field from both theory and experiments. The objective of this short comment is to clarify several of these issues.

## 1. Introduction

This short letter serves as a rebuttal to a recent article by Schmelzer and Tropin [1], which was recently published in this journal. This is not meant to be a comprehensive listing of all issues in that article. Rather, it is meant to address some of their main points criticizing modern ideas in glass science.

## 2. Definition of “Glass”

Much of the content of Schmelzer and Tropin’s paper is devoted to an attack on our recently proposed modern definitions of “glass” [2]. The first definition presented in our paper is meant for the general public and non-experts in the field: “*Glass is a nonequilibrium, non-crystalline state of matter that appears solid on a short time scale but continuously relaxes towards the liquid state*”. We also proposed an alternative, more detailed definition for use by advanced students and professionals in the field: “*Glass is a nonequilibrium, non-crystalline condensed state of matter that exhibits a glass transition. The structure of glasses is similar to that of their parent supercooled liquids* (*SCL*)*, and they spontaneously relax toward the SCL state. Their ultimate fate is to solidify, i.e., crystallize*”.

While our definition stresses the hybrid nature of the glassy state, combining both solid-like and liquid-like features, Schmelzer and Tropin favor older definitions from the 1920s–1930s, where glass is treated as strictly a solid. As we explain in Ref. [2], the glass transition is a kinetic transition involving a gradual freezing-in of the supercooled liquid (SCL) state to a solid-like glassy state. However, this freezing-in is only temporary, since the glass continuously relaxes toward the liquid state over a long time. This relaxation has been demonstrated in numerous silicate [3], organic [4,5,6,7], chalcogenide [8], and metallic [9] systems.

## 3. On Greek Philosophy

Schmelzer and Tropin [1] continue their argument by drawing upon the pre-Socratic Greek philosopher, Heraclitus, who is invoked five times in their article. However, the work of Heraclitus has nothing to do with glass. While Heraclitus certainly observed (liquid) rivers flowing in his time scale, we wonder how Heraclitus measured the flow of crystals during his lifetime, 535–475 BC? Where are his experimental results published?

## 4. Glass Flow vs. Relaxation

Regarding glass flow and relaxation, Schmelzer and Tropin [1] write:

“The fact that predominantly glasses **flow** with a perceptible rate on relevant time scales only in a certain temperature range and not beyond is well known in glass technology [24], as is clearly formulated also for example by Tammann [40,41] and is already given in the title of his well-known paper by Tool [53]. Of course, for certain applications, flow processes have to be taken into consideration as is well known already from the work of Kohlrausch, Weber, Williams & Watts, Adams & Williamson, Eyring & Tobolsky (see, e.g., [54]) starting around 1850. However, such possible flow processes under certain conditions have not been considered as essential by Tammann and Simon in their definition of glass. Such a point of view, that flow processes may be neglected in most applications for relevant times scales, has also been clearly expressed by one of the authors of Ref. [52] in Reference [55,56]. For example, in Ref. [55] it is noted that “*window glasses may flow at ambient temperature only over incredibly long times, which exceed the limits of human history*”. The flow processes considered in Ref. [55,56] are primarily the response to external fields and are governed by viscosity. However, the viscosity and structural relaxation time are uniquely correlated as noted also in Ref. [55,56], where the analysis is performed widely in terms of relaxation times.”

References [55,56] in [1] (Refs. [10,11] in this letter) are indeed authored by one of us (Zanotto). In these two articles, we estimated the average *relaxation* times (in the absence of any external stress)—*not* the flow times under stress—of GeO_2_ glass and a commercial soda-lime-silica glass at room temperature, i.e., approximately 530 °C below their laboratory glass transition temperatures. The calculated relaxation times are indeed astronomical, but they are still **finite!**

More recently, Mauro and co-authors [12] used an improved model and new experimental viscosity measurements to calculate the times needed for a real medieval glass—the Westminster Abbey cathedral glass—to relax (under zero external stress) and also to flow under gravity. The results are billions of years for both times. Moreover, the concept of a single relaxation time below *T_g_* has been challenged on several fronts; in particular, multiple relaxation mechanisms have been demonstrated for low molecular weight systems and polymers [13], chalcogenides [8], and metallic glasses [14,15].

Schmelzer and Tropin [1] have also confused the concepts of flow and relaxation in our definition. Our shorter definition reads [2]: “*Glass is a state of matter that appears solid on a short time scale but continuously relaxes towards the liquid state*”. Glasses are thermodynamically unstable against the supercooled liquid (SCL) and spontaneously relax—even in the absence of external stress—towards the SCL state. Solids do not undergo spontaneous relaxation. They may creep or flow (by different mechanisms than glasses) under external stress.

Schmelzer and Tropin continue [1]:

“In Ref. [52], Zanotto and Mauro discuss Simon’s definition of glass as a freezing-in process. Referring to Ref. [30] and presenting Simon’s point of view in the form that “*glass is a rigid material obtained from freezing-in a supercooled liquid in a narrow temperature range*”, they further state that “*it is not clear if he intended to convey the same meaning we are using here* (*frozen* = *a temporary state*)”. However, the meaning Simon assigned to his statement of freezing-in is clearly reflected in Ref. [32]. In free translation, it sounds as though freezing-in at *T_g_* does not imply that below *T_g_* relaxation processes are excluded. However, any such structural transformations proceed already slightly below *T_g_* with such large time scales that the suggestion of a permanent arrest of such structural changes is completely substantiated ([32], p. 223); or, as stated by Davies and Jones ([57], p. 375), “*Simon pointed out that as a glass is cooled through its transformation temperature the molecular diffusion which is necessary to effect the appropriate change in configuration is increasingly inhibited and finally becomes practically impossible*”. This interpretation is fully in line with Ref. [55,56] but not with the revised definition of a glass given in Ref. [52]”.

The relaxation times of a glass strongly depends upon the chemical composition (e.g., the liquid fragility), the temperature (i.e., how far below *T_g_* the material is) and the entire thermal history (i.e., the degree of thermal disequilibrium). For small undercoolings below *T_g_*, the relaxation times (and flow under stress) will be only minutes or hours. However, if one is studying his/her glass well below the *T_g_*, the relaxation and flow times will be extremely long, as shown in Ref. [3,4,5,6,7,8,9,10,11,12,13,14,15,16,17]. But, in the end, they all relax and flow at any temperature above the absolute zero. This is a requirement for a nonequilibrium, thermodynamically unstable system with finite thermal energy.

## 5. Viscosity

Schmelzer and Tropin [1] draw upon the notion of divergent viscosity in glass-forming liquids, writing:

“Moreover, the viscosity of glass-forming melts increases dramatically with decreasing temperature [24,58]. One of the relations describing it with a sufficiently high degree of accuracy for most applications is the Vogel–Fulcher–Tammann equation widely employed in glass science [59]. This equation results in a divergence of the viscosity at finite values of temperature, denoted as Vogel temperatures. Whether the viscosity will really diverge or not is a matter of intensive debate; it cannot be established by direct experimental investigations restricted to maximum values of viscosity less than 10^18^ Pa·s. In any case, a variety of models of the vitreous state lead to the confirmation of such a conclusion. However, once the viscosity diverges, the structural relaxation time also diverges. Glasses at temperatures below the Vogel temperature are then excluded from the vitreous state by the above-mentioned definition.”

The Vogel-Fulcher-Tammann (VFT) was proposed in the 1920s as an empirical function to describe the viscosity of liquids and supercooled liquids [18]. However, the VFT equation does not describe the viscosity of the nonequilibrium glassy state, which is typically many orders of magnitude less than that of the corresponding supercooled liquid at the same temperature. This is well known, both through experimental measurements and basic theory [16,17]. Second, the divergence predicted by the VFT equation (for supercooled liquids, not glasses!) is an artifact of the particular form of this empirical equation. Since the 1920s, several more successful and physically derived models have been proposed that do *not* predict such divergence and have been shown to provide a dramatically improved description of liquid viscosity at low temperatures. Unfortunately, this work is not cited by Schmelzer and Tropin. Readers interested in a review of the modern understanding of the viscosity of glass-forming systems are referred to Ref. [17].

## 6. Crystallization

Schmelzer and Tropin [1] argue against the reality of glass crystallization, writing:

“Further extending their modification of the definition of glass, Zanotto and Mauro propose to include into the definition of glass the statement, “*Their ultimate fate, in the limit of infinite time, is to crystallize*”. However, even if this statement would be true, it seems to us not to be reasonable to include such a statement into the definition, as it does not supply any additional information as to what glasses are. In addition, if at all, crystallization proceeds at a perceptible rate for states below the glass transition range also only at time scales exceeding the limits of human history.”

However, glasses will always relax and then crystallize. Moreover, in certain cases, they can even crystallize before full relaxation [2]. As for relaxation, the actual crystallization time depends upon the chemical composition of the material and the temperature. If one is testing a glass a few degrees below its *T_g_*, these processes will take only minutes or hours; if one is working well below *T_g_*, say at room temperature for a typical oxide glass, crystallization will take longer than the age of the universe. To give a few examples, in the Zanotto laboratory we have crystallized lithium diborate glass and a diopside (CaO·MgO·2SiO_2_) glass, 30–50 °C below their respective *T_g_* in less than 3 months [19]. Crystallization (or “devitrification”) is also a well-known critical problem for the glass industry [20,21].

As shown in Ref. [20], even the most stable oxide glasses known so far, B_2_O_3_ and albite, will crystallize below their *T_g_* with adequate heat treatments. The crystallization times are indeed very long well below *T_g_*, but they are **finite**. Even glasses that do not exhibit congruent crystallization will still crystallize incongruently in the limit of long time—in this case the primary devitrification phase corresponds to the crystal having the minimum Gibbs free energy in the phase diagram of the system. Finally, we should mention that atactic polymers do not crystallize because they are unstable artificial materials that degrade (their molecular weight dramatically decreases) upon treatment in a temperature range (somewhat above *T_g_*) where crystal nucleation and growth would be typically observed in laboratory time scales.

In our opinion, this part of our new definition is very important because it reinforces the fact that glasses are not true solids: They only solidify at the end of their lives, when they crystallize. Our message is that all of the dynamic processes that take place above the laboratory *T_g_* (ionic diffusion, viscous flow, relaxation, liquid-liquid phase separation, crystal nucleation and growth, etc.) also take place below the *T_g_*. It only takes longer as the temperature drops. 

## 7. Broken Ergodicity and Entropy

Regarding the concepts of broken ergodicity and the configurational entropy of glass, Schmelzer and Tropin write [1]:

“Our conclusion is that the treatment of vitrification as a process of continuously breaking ergodicity with entropy loss and a residual entropy tending to zero in the limit of zero absolute temperature is in disagreement with the absolute majority of experimental and theoretical investigations of this process and the nature of the vitreous state. This conclusion is illustrated by model computations.”

Unfortunately, their model does not account for broken ergodicity (i.e., the non-equivalence of time and ensemble averages of properties due to the long timescales involved with glass science), which we argue is the single most fundamental, defining feature of the glassy state [22,23,24]. One cannot accurately model the thermodynamics or statistical mechanics of the glassy state without accounting for its non-ergodic nature. This was demonstrated by Mauro et al. [25] who derived model-free equations for the entropy of glass considering both ensemble and time averages. A finite residual entropy is present only in the ensemble-average formulation, which would also predict a non-zero heat capacity of glass at absolutely zero temperature. In contrast, the time-average formulation yields zero entropy at absolute zero, since the glass is confined to a single microstate, and all of its properties are an average over this one microstate alone. Without fluctuations, the time-average formulation yields zero heat capacity in the limit of absolute zero temperature, which is consistent with experimental results [25]. While it is possible to construct a model to obtain any result that one desires, as Schmelzer and Tropin have done [1], the ultimate truth must come from experiments, where there is no doubt that glasses are confined to a single microstate in the limit of absolute zero, and hence have zero heat capacity and zero entropy, consistent with the Third Law of Thermodynamics [25].

## 8. Conclusions

We have given arguments against several ideas promoted by Schmelzer and Tropin [1] regarding viscous flow, relaxation, crystallization, and entropy of supercooled liquids and glasses. We emphasize that recent theoretical and experimental understanding of glass-forming systems must be considered with respect to each of these topics. New advances in the applications of glass can only be made by taking advantage of the improved understanding of the fundamental materials processes driving their properties and behavior [26]. It is, therefore, essential to embrace progress in glass science on both experimental and theoretical fronts.

We are thankful to Daniel R. Cassar, Marcio, L.F. Nascimento, and Raphael, M.C.V. Reis for their critical comments and suggestions. 
